# The effect of size on the strength of FCC metals at elevated temperatures: annealed copper

**DOI:** 10.1080/14786435.2016.1224945

**Published:** 2016-08-30

**Authors:** Jeffrey M. Wheeler, Christoph Kirchlechner, Jean-Sébastien Micha, Johann Michler, Daniel Kiener

**Affiliations:** ^a^ Laboratory for Nanometallurgy, ETH Zürich, Zurich, Switzerland; ^b^ Laboratory for Mechanics of Materials and Nanostructures, Empa, Swiss Federal Laboratories for Materials Science and Technology, Thun, Switzerland; ^c^ Structure and Nano-/Micromechanics of Materials, Max-Planck-Institut fur Eisenforschung GmbH, Dusseldorf, Germany; ^d^ Department of Materials Physics, Montanuniversität Leoben, Leoben, Austria; ^e^ UMR CNRS-CEA SPrAM, Institute Nanosciences and Cryogenics, Université Grenoble Alpes, Grenoble, France; ^f^ CRG-IF BM32 Beamline at the European Synchrotron (ESRF), Grenoble, France

**Keywords:** Size effect, copper, high temperature deformation, µ-Laue diffraction

## Abstract

As the length scale of sample dimensions is reduced to the micron and sub-micron scales, the strength of various materials has been observed to increase with decreasing size, a fact commonly referred to as the ‘sample size effect’. In this work, the influence of temperature on the sample size effect in copper is investigated using *in situ* microcompression testing at 25, 200 and 400 °C in the SEM on vacuum-annealed copper structures, and the resulting deformed structures were analysed using X-ray μLaue diffraction and scanning electron microscopy. For pillars with sizes between 0.4 and 4 μm, the size effect was measured to be constant with temperature, within the measurement precision, up to half of the melting point of copper. It is expected that the size effect will remain constant with temperature until diffusion-controlled dislocation motion becomes significant at higher temperatures and/or lower strain rates. Furthermore, the annealing treatment of the copper micropillars produced structures which yielded at stresses three times greater than their un-annealed, FIB-machined counterparts.

## 1. Introduction

As the length scale of mechanical test specimens decreases to the micron scale and below, an anomalous strengthening trend is usually observed with decreasing size [1–4]. This has been long observed in the form of Hall–Petch strengthening [5,6] for decreasing grain size, and more recently as an indentation size effect [7] with increasing hardness and decreasing indentation depths. The extent of this size-dependent strengthening is typically described using an empirical power law relationship [2]:(1) 
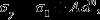



where *σ*_*y*_ is the yield strength, *σ*_0_ is the bulk strength, *A* and *n* are constants and *d* is the characteristic size being interrogated. Micro-compression testing on pillars machined using focused ion beam (FIB) techniques was originally pioneered by Uchic et al. [8] in order to avoid the indentation size effect phenomenon for extraction of constitutive parameters for deformation. Instead, it was discovered that the size effect was a more general phenomenon than expected. Some efforts to relate the length scale of deformation in a wide range of loading and test geometries are now making headway [9–13].

The majority of size-dependence studies on materials have focused primarily on metals, particularly those with the face-centred cubic (FCC) crystal structure: Pt [14], Ni [8,15–18], Cu [19–23], Au [24–30], Ag [31] and Al [32–38]. In all these studies, a similar size dependence was observed, where the power-law exponent, *n,* ranged between −0.6 and −1.0. Furthermore, if the differences in Burgers vector and shear moduli are compensated for, then the characteristic value for *n* was found to collapse towards ~ −0.7 [1,3,4,11].

The size-dependence of body-centred cubic (BCC) metals has been observed to be more variable [29,39]. This has been rationalised by taking into account the contribution of the lattice resistance, which scales with the ratio of the test temperature to the critical temperature (*T*_c_). The lower size dependence observed in the BCC micropillars was attributed to the decreased mobility of the screw dislocations [39], which scales with the critical temperature [40]. This has recently been experimentally verified by systematic testing of size effects of Mo [41], Ta and W [42] at elevated temperatures. Similar behaviour has also been observed using high temperature nanoindentation on W and Cr [43].

Despite the significant role of temperature in the mechanical properties of materials and demonstrated relationships for many size-dependent materials [41,42,44], very few investigations of the temperature dependence of the size effect in FCC metals are yet to be performed. This is due to the limited availability of micromechanical testing systems capable of performing such precision testing at elevated temperatures under stable conditions [45]. However, recent advances are constantly pushing the accessible temperature ranges both higher (~1000 °C [46,47]) and lower (−150 °C [48,49]).

If one considers the effective homologous temperatures (*T*/*T*_m_, where *T*_m_ is the melting temperature) of the various FCC metals which have been tested at 25 °C (Ni – 0.172*T*_m_, Cu – 0.220*T*_m_, Au – 0.223*T*_m_ and Al – 0.319*T*_m_) in a similar fashion to the way in which Schneider et al. [39] addressed the temperature dependence of BCC metals, it might be expected that the size dependence of FCC metals would have negligible temperature dependence, as these metals were found to have similar size dependence in the limited range between 0.17 and 0.32 *T*_m_. Using elevated temperature nanoindentation, Franke et al. [50] investigated the indentation size effect in copper up to 200 °C (0.348 *T*_m_) and observed a significant decrease in magnitude at elevated temperature, in contrast to microcompression results of Al at similar homologous temperatures. Therefore, the aim of the present paper is to systematically investigate the plasticity of FCC pillar structures at the micron and sub-micron regimes, for the first time, at elevated temperatures up to 400 °C (0.495 *T*_m_).

## 2. Experimental procedures

### 2.1. Sample preparation

5 mm × 5 mm wedges were prepared from single crystal copper with a (1 0 0) orientation (MaTecK GmbH, Juelich, Germany), using mechanical and electrochemical polishing [51]. The thick, bottom portions of the wedges were then mechanically clamped within a standard half inch diameter stub, producing a freestanding 4 mm portion of the wedge. This approach is intended to reduce the lateral stiffness of the testing equipment, but ensures good thermal contact between the stub and the sample. In order to produce pillars with a uniform cross-section across their entire height, FIB machining using a Zeiss 1540 XB (Zeiss, Oberkochen, Germany) was performed perpendicular to the wedge to produce pillars with square cross-sections. The final shaping was performed under grazing ion incident with a milling current of 100 pA to reduce the ion damage [52,53]. The top surface was milled in a final step being inclined by 1.5° to accommodate for tapering. Three wedges were produced with pillars having side lengths of approximately 0.4, 1, 2 and 4 μm and an aspect ratio of 2.5:1 [54]. Three pillars were produced for each condition, and a different wedge was used to perform tests at each temperature. High resolution scanning electron microscopy (SEM) pictures were taken from different viewing directions before and after the deformation experiments using a Zeiss 1525 SEM.

### 2.2. Mechanical testing

Compression of the pillars was performed at temperatures of 25, 200 and 400 °C using a modified Alemnis SEM indenter inside a Zeiss DSM 962 SEM [55,56]. The high vacuum (5 × 10^−5^ mbar) of the SEM chamber both simplifies the heat transfer situation inside the system and prevents oxidation of the samples, which is a significant consideration for copper. Prior to compression, the samples were heated to 600 °C and annealed for 1 h *in situ* in the system to remove most of the dislocations introduced by the FIB machining procedures [57]. After this heat treatment, the samples were cooled to their desired test temperature. In order to prevent heat flow and thermal drift during testing, the temperatures of the thermally calibrated indenter and sample were carefully matched prior to compression using direct temperature measurements in contact with areas of the wedges near the pillars [58]. This produces an isothermal testing environment with temperature accuracy on the order of 1 °C within this temperature range. Compressions were performed at a constant strain rate of 1 × 10^−3^ s^−1^ by applying displacement rates appropriate to the sample dimensions. Engineering stress–strain curves were determined using the measured load over the original top surface area of the pillars and using the displacement relative to the pillar’s height after correcting for compliance and pillar sink-in [56].

### 2.3. Microdiffraction Laue (μLaue) characterisation

**Figure 1.**
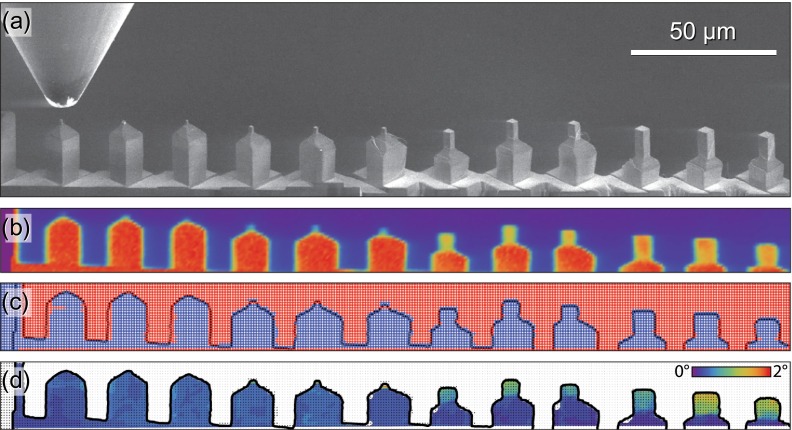
(colour online) Overview of sample tested at 200 °C in (a) SE microscopy, (b) X-ray Cu K_α_ fluorescence map, (c) map showing positions where Laue patterns were successfully indexed in blue and (d) the misorientation determined by μ-Laue diffraction.

The post-mortem μLaue experiments were performed at the French CRG-IF BM32 beamline at the European Synchrotron Radiation Facility in Grenoble, France. A polychromatic X-ray beam with a spectral range of 5–22 keV was focused to 500 × 500 nm spot size at full width at half maximum. Prior to scanning, the set-up was calibrated using an unstrained Germanium wafer. Subsequently, Laue patterns were acquired using a Rayonix CCD camera with 2048 × 2048 resolution, a pixel size of 79 μm and an exposure time of 0.2 s. Simultaneously, the X-ray fluorescence emitted by the sample was recorded by an energy dispersive X-ray detector (Röntec GmbH, Germany) – Figure 1(b). The mesh scans were performed with a step size of 1 μm. Figure 1(c) shows the individual mesh positions where at least one Laue pattern could be successfully indexed as blue dots. Data analysis, i.e. set-up calibration as well as indexing and refinement as presented in Figure 1(d), was performed by the software package LaueTools [59] with post-treatment in Mathematica®.

## 3. Results

### 3.1. Mechanical behaviour

**Figure 2.**
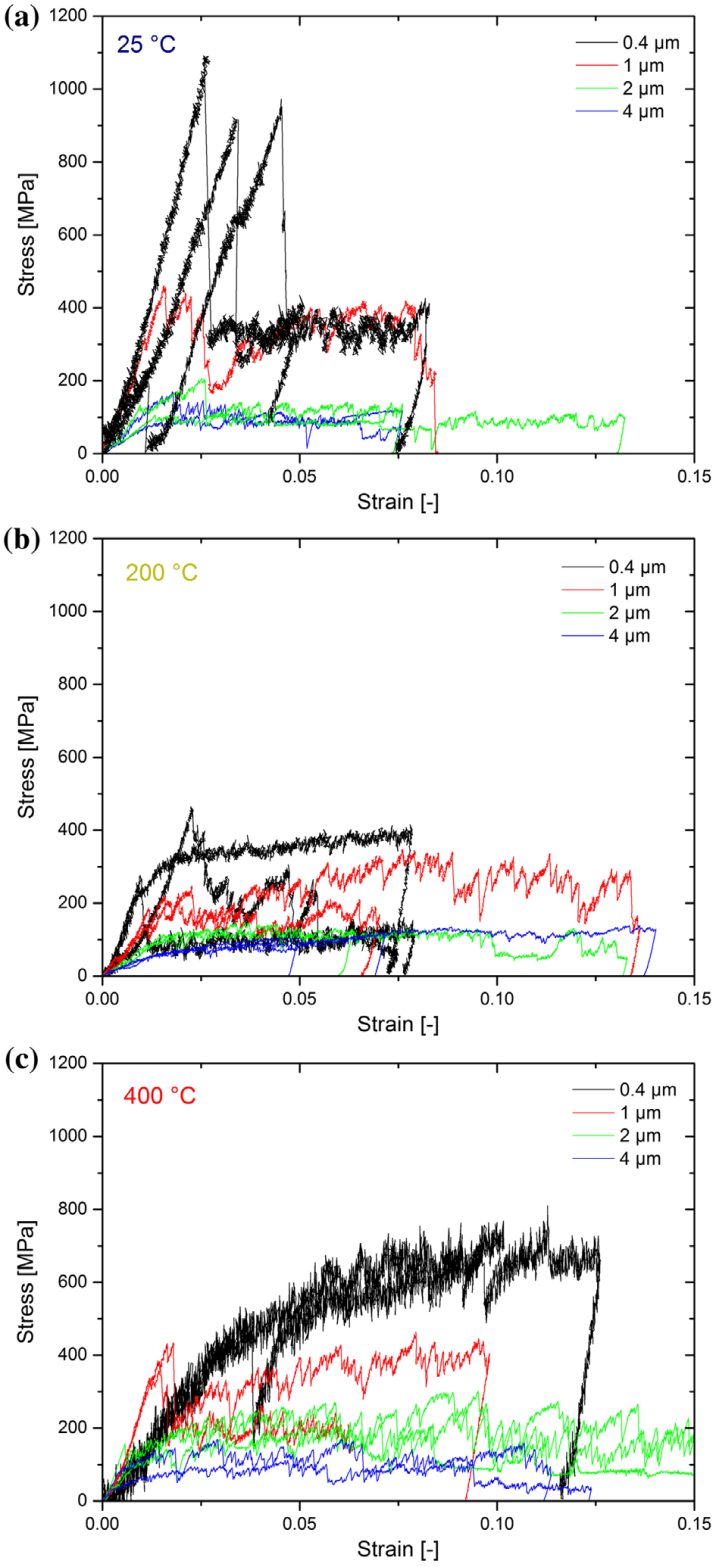
(colour online) Stress–strain curves from (1 0 0) oriented Cu pillars compressed at (a) 25, (b) 200 and (c) 400 °C with results grouped by approximate average pillar edge length.

The stress–strain behaviour of the pillars is summarised in Figure 2. At ambient temperature, the flow curve exhibits an upper yield point, consistent with the annealing treatment having effectively removed the majority of the FIB-induced dislocations – forcing yielding to occur via nucleation of new dislocations at much higher stresses [57]. After this, deformation proceeds by slip at much lower stresses, so-called easy glide. At higher temperatures, this upper yield point phenomenon is less frequently observed.

**Figure 3.**
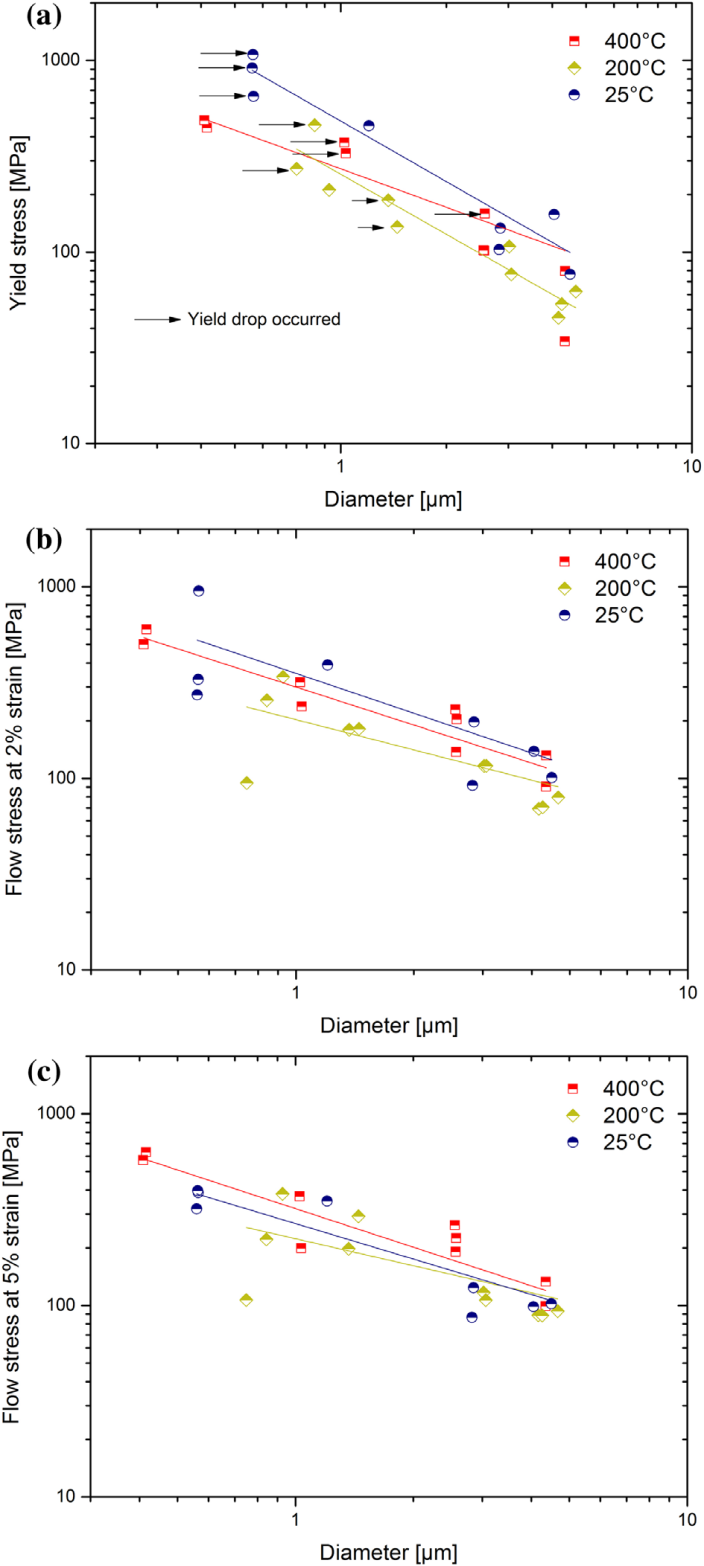
(colour online) Yield behaviour for different criterions: (a) initial yield stress, (b) flow stress at 2% and (c) 5% strain as a function of equivalent pillar diameter for each test temperature.

The variation in the yield strengths and the nucleation behaviour in the samples tested at different temperatures can be attributed to two different factors: variability in the annealing effectiveness for different wedges or differing misorientation of the different wedges tested at each temperature. Some insight into this can be provided by considering the temperature- and size-dependent strength at different yield/flow stress criterions, as provided in Figure 3. The first criterion, the yield stress, was designated as the stress where the first significant change in stiffness was observed. The characteristic length scale of each rectangular pillar in Figure 3 is represented by the diameter of a circle having identical area.

One surprising observation that can be immediately made from Figure 3 is that the values from 400 °C tend to be slightly higher than those at 200 °C. However, the values from all the temperatures are fairly consistent within their own experimental scatter. The power law slopes are fairly similar between all the temperatures for each strain condition, but the slopes of the yield stress fits, Figure 3(a), are higher than those in the flow stress criteria plots, Figure 3(b) and (c).

Results from the extracted normalised work hardening rates from the stress–strain behaviour from Figure 2 were found to be inconclusive due to significant scatter in the measured rates. A slight trend towards increasing work hardening at smaller sizes was observed, as seen previously [21]. However, there was no clear trend with temperature, and variation in misalignment between the samples, as will be discussed later, is expected to be the controlling parameter on the work hardening performance [60].

### 3.2. μLaue characterisation of deformation behaviour

All samples deformed at 200 and 400 °C were analysed by *post mortem* X-ray μLaue diffraction. The acquired diffraction patterns typically consisted of 25 high-intensity Laue spots, which could all be correctly assigned to copper reflections. Thus, within the resolution of this experiment, no evidence of copper oxide was found. If FCC Cu_2_O or monoclinic CuO had been present, even in an epitaxial growth orientation, it would have created additional reflections. This is not definitive proof of the absence of thin layers of copper oxides, as the high intensities of the thick copper sample do not allow for the required acquisition times to observe any small amount of crystalline oxide. An amorphous oxide layer would be invisible to Laue. However, the thickness of the oxide films appears to be negligible, and any small amount of oxide present should not significantly contribute to the mechanical performance of the samples.

**Figure 4.**
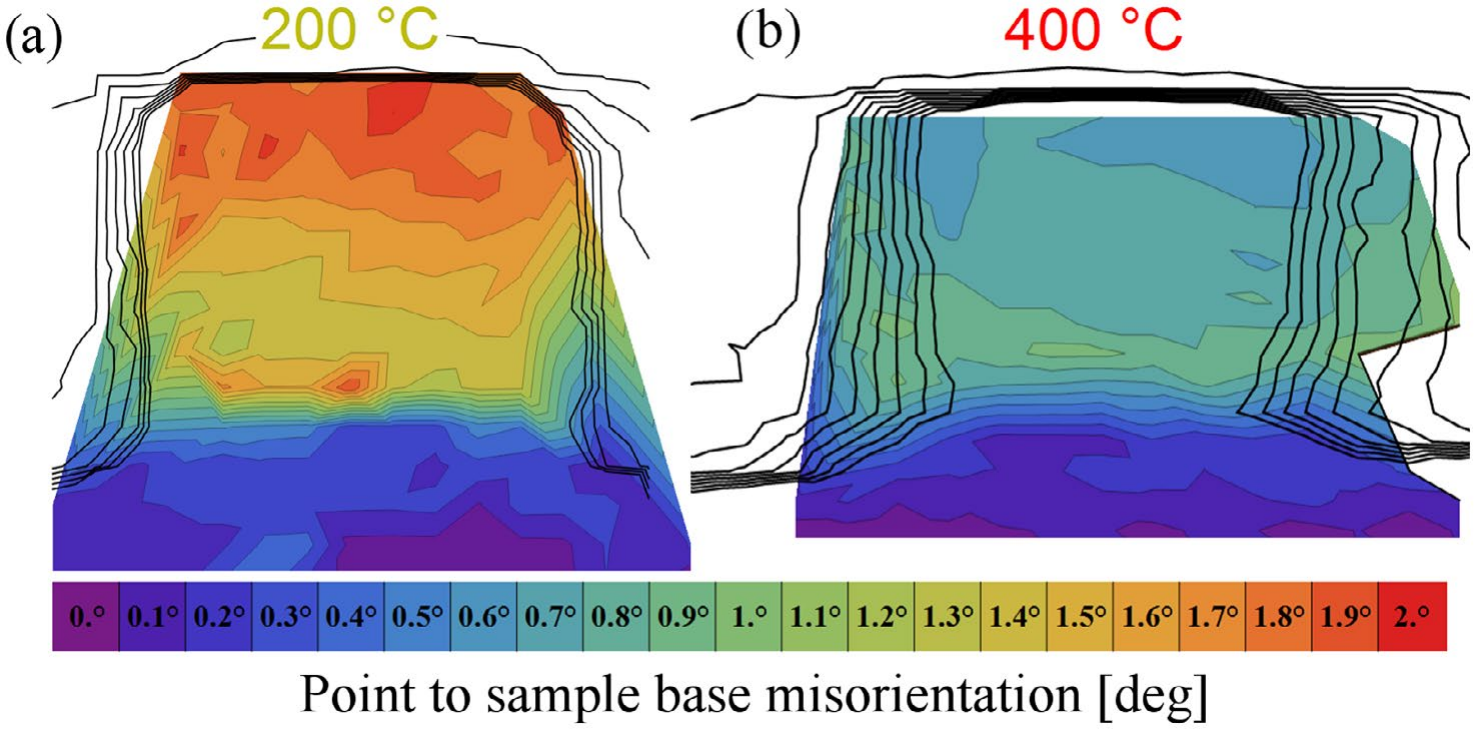
(colour online) μLaue misorientation maps exemplarily shown for a 4 μm pillar deformed (a) at 200 °C and (b) at 400 °C. The black contour lines show the superimposed fluorescence signal, representing the pillar geometries. The figures show a clear orientation gradient from the bottom to the top, but relatively small lateral orientation variations.

**Figure 5.**
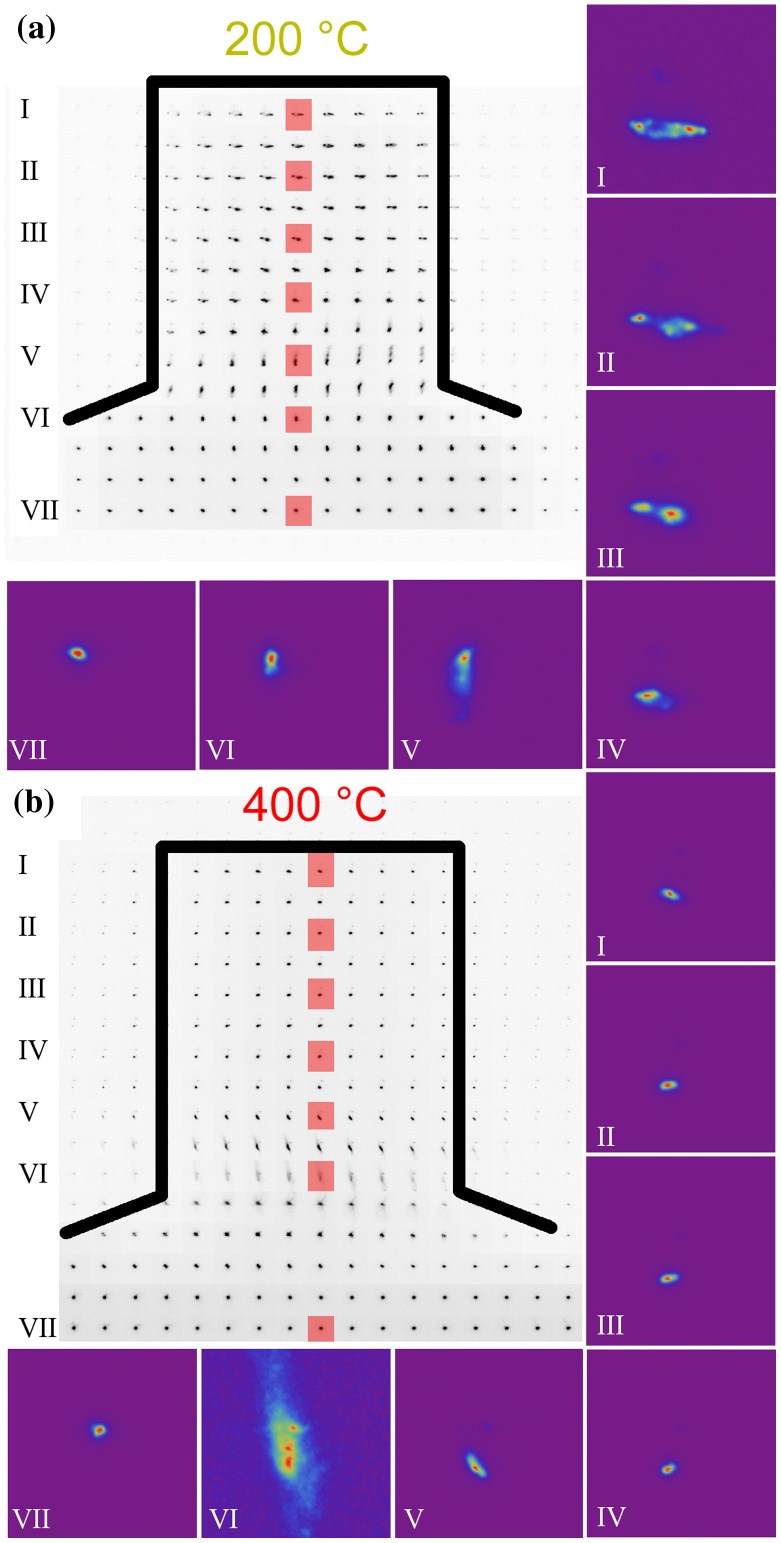
(colour online) Composite image of the (1 1 1) Laue spot recorded across 4 μm pillars deformed (a) at 200 °C and (b) at 400 °C. The magnified insets (I–VII) present the spot evolution of the diffraction peak from top to bottom in the pillar centre for each sub-figure. The corresponding positions are marked in the composite image by red rectangles. Refer to text for details.

Representative μLaue misorientation maps, as computed from the crystallographic orientations obtained by LaueTools, are shown in Figure 4. The 200 °C pillar in Figure 4(a) shows a continuous increasing gradient in misorientation from the sample base towards the top. The point-to-base misorientation thereby reaches a maximum of almost 1.6° for a 4 μm pillar. By examining the diffraction peak shape of the (1 1 1) reflection, as presented in Figure 5(a), it is evident that most parts of the pillar show extensive peak broadening which suggests the storage of geometrically necessary dislocations (GNDs). Across the entire width of the pillar the presence of at least one additional Laue spot, which is separated by up to 1°, can be noted. This behaviour was also previously observed [60] in misaligned samples, where comparable diffraction patterns were seen even though the straining direction was significantly different.

In comparison to the 200 °C pillar, the pillar deformed at 400 °C shows several distinct differences. On the one hand, the point-to-base misorientation reaches a maximum (~0.9°) at half the sample height, rather than the pillar apex – Figure 4(b). Further approaching the sample top reduces the point-to-base misorientation, evidencing that the alignment was better in the 400 °C specimen with an average misorientation of only 0.6°. On the other side, the diffraction peak shape of the (1 1 1) spot, as presented in Figure 5(b), significantly differs from the specimen tested at 200 °C. Besides some streaking consistent with the storage of GNDs close to the sample base, the 400 °C specimen does not show any pronounced streaking within the entire pillar volume. This is similar to previous *in situ* findings in compression [60] and tension samples [61] of 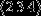-oriented single crystals. In contrast to the aforementioned work, any misalignment in the pillars tested in this work should be accommodated more easily, since the symmetric slip orientation of the [1 0 0]-oriented pillars allows a large number of possible slip systems to accommodate and stabilise the plastic deformation at greater strains. Furthermore, we do not see the storage of a distinct slip system at the sample base, instead a blurry (1 1 1) Laue spot with three sub-peaks is observed – Figure 5(b) VI. This is most likely caused by the simultaneous activation of several slip systems, as would be expected for this symmetric crystal orientation.

The observations presented on the two pillars shown here apply for all pillars of the two specimens investigated at elevated temperatures. However, due to the strong strain gradients in smaller pillars at the top, only the largest pillars of a wedge were used to evaluate corresponding misalignments. From the Laue data, it can be concluded that the wedge deformed at 200 °C was misaligned by 1.3° ± 0.5° and the 400 °C wedge by 0.4° ± 0.2°. In all cases, the data show negligible misalignment in the 400 °C wedge, which does not cause extensive storage of GNDs, but peak streaking and sub-grain formation in case of the wedge deformed at 200 °C.

### 3.3. Scanning electron microscopy characterisation

**Figure 6.**
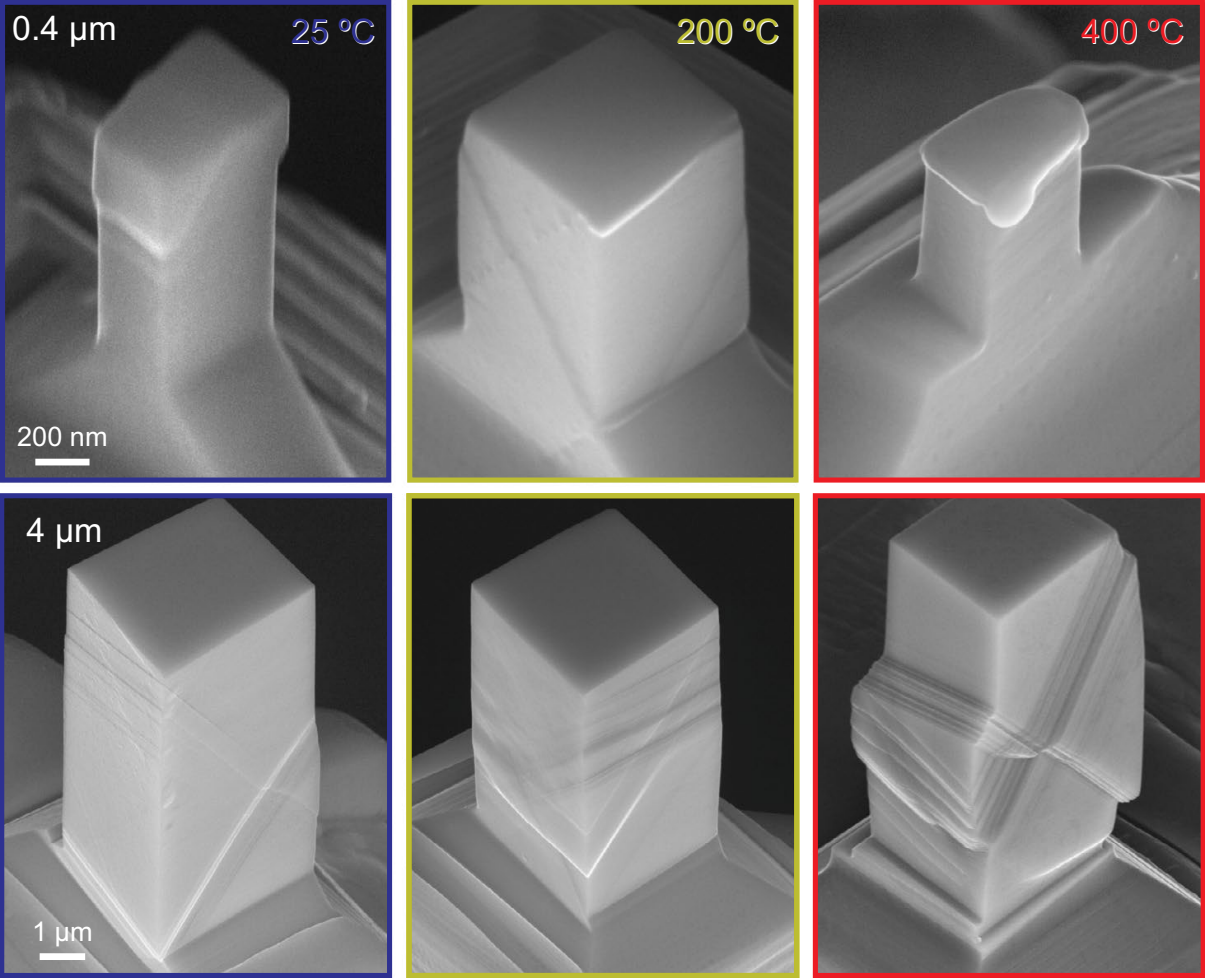
(colour online) Overview of specimen deformation from SEM micrographs of the smallest (0.4 μm) and largest (4 μm) pillars from each testing temperature.

As the resolution of the microscope used for *in situ* compression was limited, the deformed pillars were characterised using high-resolution SEM after compression. Representative results in terms of the two extremes in size as a function of temperature are shown in Figure 6 for a 0.4 and a 4 μm pillar, respectively. At the smaller sizes, diffusion is more important, as evidenced by the increasingly rounded edges at the slip offsets. This is not too surprising, as the test temperatures approach half the melting point of the material, where surface diffusion sets in [62]. Furthermore, the Ga ions implanted during FIB machining may have alloyed with the near surface to reduce the melting point of the material, as observed previously for FIB prepared Mg pillars [63]. However, the high-strength values observed suggest that diffusion only affects the appearance of the surface features on the deformed samples and does not play a significant role during the deformation. Notably, the pillars did not change shape or melt during the 1 h annealing at 600 °C or prior to deformation at elevated temperatures, contrary to what was previously observed for gold pillars tested at ~600 °C, corresponding to 0.65*T*_m_ [64].

For both sample sizes, slip offsets are more localised at ambient temperature. As the testing temperature increases, the slip offsets become more distributed throughout the height of the pillars. This suggests that more dislocation sources are activated at elevated temperatures. This enhanced nucleation at elevated temperatures also correlates with the pronounced upper yield peaks more commonly observed at the lower test temperatures in Figure 2. In the larger pillars tested at 400 °C, some bulging was observed in the lower portion of several of the pillars. This might be diffusion related, but Laue results (Figure 5(b)) suggest that this is may be attributed to the simultaneous activation of several slip systems.

A distinct variation in slip systems is observed in the sample deformed at 200 °C. In specimens tested at 25 and 400 °C, surface slip offsets are observed on the 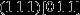 and 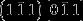 slip systems, which can be most clearly seen on the right faces of the 4 μm pillars in Figure 6 as sets of opposing diagonal lines. In contrast, in the samples deformed at 200 °C, the slip offsets are observed to correspond to the 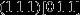 and 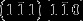 slip systems. The 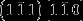 slip system can be observed as a horizontal line on the right face of the 4 μm pillar deformed at 200 °C in Figure 6. This variation is consistent with the ~1.8° misalignment measured in the previous section using μLaue diffraction.

## 4. Discussion

**Figure 7.**
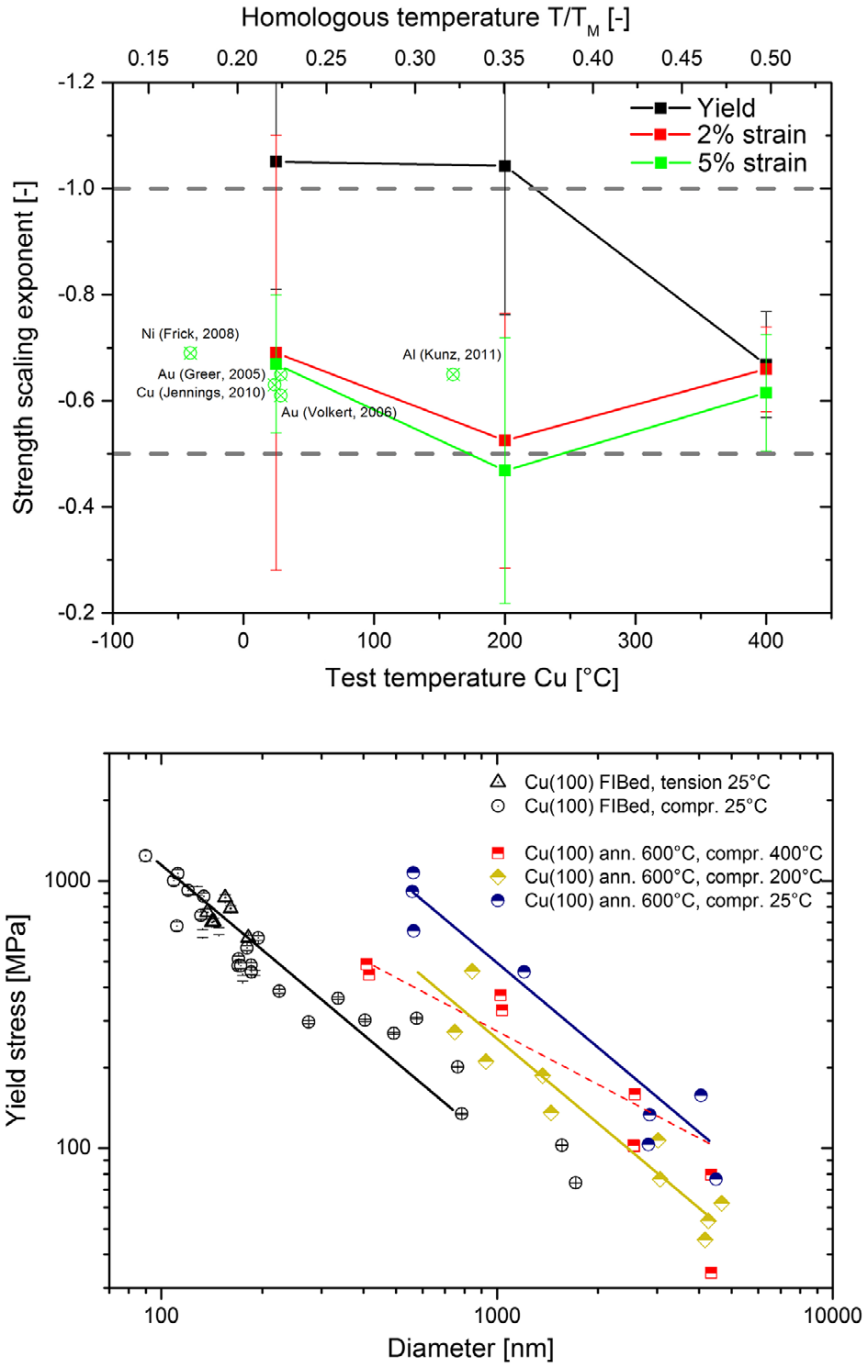
(colour online) (a) Size effect strengthening exponent for the annealed Cu(1 0 0) as a function of temperature for various yield criteria, including exponents from various FCC metals [17,23,24,26,36] at 5% strain as a function of homologous temperature (upper axis). (b) Size dependent strength as compared to literature data on FIB-prepared Cu [20,21].

The size effect exponent from each of the fits in Figure 3 is given as a function of temperature in Figure 7(a). For both of the flow criterions, the scaling exponent is observed to be constant within the significant scatter at ~ −0.65, very much alike in previous reports [1,3,4]. The yield behaviour shows a slightly different trend. At the two lower temperatures, the value is consistent at *n* = −1, but at the highest temperature it is observed to drop to −0.65. Whether this is due to a change in dislocation source activation at high temperatures or due to misalignment is at this point not clear. However, if misalignment produced such a reduction in scaling exponent, then the results from 200 °C might be expected to be lower, since that sample showed the lowest trends in strength, which one would attribute with the highest misorientation (Figure 3). This is, however, not the case. The literature data for other FCC metals at room temperature for a 5% flow stress criterion, which produces a limited range of homologous temperatures for the different metals, is shown on the top axis. This is observed to be consistent with our room temperature experiments at 5% flow stress, and a constant, or possibly slightly decreasing, trend with increasing temperature is also observed.

From Figure 7(b), it can be seen that the strength of the annealed samples in the current work clearly lies above the as-FIB prepared Cu, particularly for the smaller samples. At increasing diameters, the difference is reduced. The strength scaling exponent was reported to be −1 from sub-micron *in situ* TEM experiments [22], in accordance with the exponents measured in the current study in the range between 0.4 and 4 μm. A scaling exponent of −1 is typically interpreted as implying that the deformation behaviour is source-limited [4,22,65]. Taking into account that the dislocation content in the pillars was modified by annealing, thereby removing the FIB surface defects and possibly all long mobile dislocation segments in the samples [57], it is not too surprising that the pillars resemble this source-limited behaviour. Similar observations were also made using 3D discrete dislocation simulations, where high initial yield points were observed when the model started with a narrow distribution of small dislocations sources. Using analytical models and MD simulations, Zhu et al. [66] calculated compressive nucleation stresses of 1.3 GPa for dislocations from a corner at this strain rate (1 × 10^−3^ s^−1^), which is very comparable to the stress drops we observed in Figure 2(a) at stresses of ~1 GPa at ambient temperature. If we apply a simple exponential extrapolation from Zhu et al.’s calculations to estimate nucleation stresses at 400 °C, then the values would be expected to be ~350 MPa, which is also quite comparable to the observed yielding in the smaller pillars in Figure 2(c). At all temperatures, after some plastic deformation, larger stable dislocation sources formed and operated at lower flow stresses [67,68], which is consistent with the behaviour observed in Figure 2.

**Figure 8.**
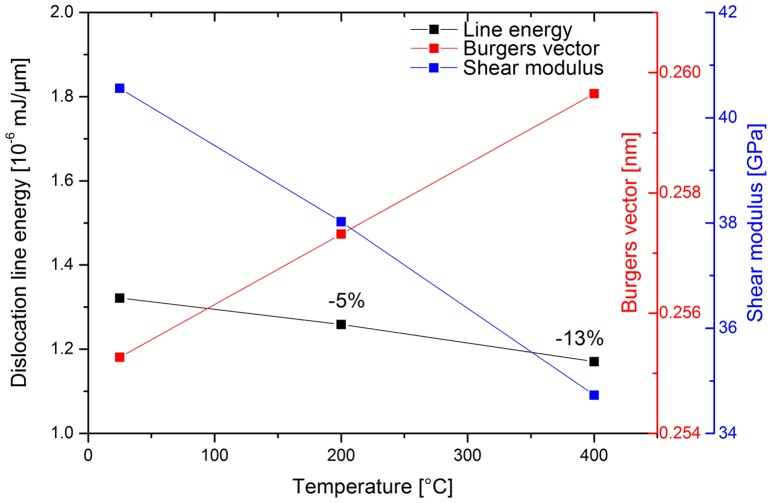
(colour online) Temperature-dependent elastic properties of Cu, calculated from [69].

Moving on to the present elevated temperature results, it is evident that, within the experimental scatter, there is a slight reduction in strength at elevated temperature (Figure 7(b)), but no pronounced change in the size strengthening effect (Figure 7(a)). This can be rationalised when considering the expected dislocation line energy as a function of temperature. In Figure 8, the temperature-dependent shear modulus, *G*, Burgers vector, *b*, and resulting line energy, *Gb* ^2^, are shown, calculated from [69]. Obviously, *G* and *b* exhibit opposite trends with increasing temperature. However, the resulting reduction in line energy is only ~5% at 200 °C and 13% at 400 °C, respectively, which based on the limited experimental data has to be considered as within the scatter.

It is worth mentioning that there was no significant decrease in flow stress with temperature observed up to 400 °C – Figure 3. This is in fairly good agreement with the data from Hirsch and Warrington [70], who only found a 10% drop in flow stress between their values at 0 and 400 °C in OFHC copper (50 μm grain size). However, this is in stark contrast to the results of Smolka et al. [71] and Wimmer et al. [72–74], who both tested polycrystalline micro-samples and observed a much stronger drop in yield strength for some microstructures. However, the results of Wimmer [74] suggested a brittle failure at the grain-boundaries due to segregation of impurities, mostly of sulphur. This indicates that the role of grain boundaries may dominate the temperature dependence of the mechanical behaviour for polycrystalline samples.

Finally, it is worth considering the possible influence of diffusion on the deformation behaviour. Comparing our results for 5% flow stress and a strain rate of 1 × 10^−3^ s^−1^ to the deformation map of coarse grained pure Cu with 100 μm grain size [75], one notes that the data for the largest 400 °C pillar falls in a regime wherein for bulk samples grain boundary-mediated Coble creep was observed. Due to the sample size effect, the smaller samples would be in the dislocation plasticity regime. Certainly, there is no guarantee that deformation mechanisms remain unaltered by the sample size reduction. In fact, for these small single crystals one would expect pipe diffusion along dislocation cores and surface diffusion to be dominant, both of which are not reflected in the Ashby maps. Moreover, the macroscopic experiments were all performed under tension, while we loaded the pillars in compression.

Nonetheless, taking a closer look at the 4 μm samples, we frequently noted an uncommon barrelling of the specimen near the base (not shown). This is the opposite of the common sample top mushroom formation known for tapered specimens [76,77]. From our present understanding, this could indicate that creep deformation contribution has contributed to the plastic deformation, but without affecting the strength level. This suggests that more systematic studies should be considered, where diffusion related deformation contribution are critically considered when testing small volumes at elevated temperatures.

## 5. Conclusions

In this work, the influence of temperature on the sample size effect in copper is investigated using *in situ* microcompression testing at 25, 200 and 400 °C in the SEM on vacuum-annealed copper structures, and the resulting deformed structures were analysed using X-ray μLaue diffraction and scanning electron microscopy. This was to allow the temperature-dependent performance of the pillar structures to be interrogated in the absence of oxidation or changes in dislocation density. The annealed copper micropillars exhibit significant strengthening due to the reduction in dislocation density within the pillars. This produced structures which yielded at stresses three times greater than their un-annealed, FIB-machined counterparts. Both μLaue diffraction and SEM indicated that the surfaces were free of oxidation, so that the mechanical results could be considered for the case of samples having free surfaces.

The primary conclusion of this work is that the magnitude of the size effect in FCC metals is measured to be constant with temperature, within the measurement precision, up to nearly half of the melting point. This is consistent with the size effect’s contribution to the material’s strength being athermal due to the limitation of dislocation source size, and the thermally activated contributions to the strength for FCC metals being negligible in comparison to the magnitude of the size effect. The possible thermal variation in the magnitude of the size effect due to dislocation line energy variation was estimated, but found to be too small to be determined within the precision of the current measurements. It is expected that the size effect will remain constant with temperature until the onset of diffusion-controlled dislocation motion becomes significant at higher temperatures and/or lower strain rates.

## Disclosure statement

No potential conflict of interest was reported by the authors.

## Funding

This work was supported by the Austrian Science Fund FWF (project number P25325-N20).
